# Cervical Spine Injuries in Older Patients with Falls Found on Magnetic Resonance Imaging After Computed Tomography

**DOI:** 10.5811/westjem.2021.5.51844

**Published:** 2021-09-02

**Authors:** Corinne H. Cushing, James F. Holmes, Katren R. Tyler

**Affiliations:** University of California, Davis School of Medicine, Department of Emergency Medicine, Sacramento, California

## Abstract

**Introduction:**

In this study we aimed to determine the rate of traumatic abnormalities on cervical spine magnetic resonance imaging (MRI) after a normal cervical spine computed tomography (CT) in older patients with ground-level falls. We hypothesized that MRI is low yield following a normal physical examination and normal CT after a ground-level fall.

**Methods:**

This was a retrospective cohort study of patients 65 years and older evaluated with a cervical spine MRI following a ground-level fall. Inclusion criteria included age 65 years and older, ground-level fall, normal cervical spine CT followed by a cervical spine MRI. We abstracted data following accepted methodologic guidelines. Patients with any focal neurological finding were considered to have an abnormal neurological examination. Imaging studies were considered to be abnormal if there was a report of an acute traumatic injury. The primary outcome was a traumatic abnormality identified on MRI. We described data with simple descriptive statistics.

**Results:**

Eighty-seven patients with a median age of 74 (interquartile range [IQR] 69, 83]) years had an MRI following a normal cervical spine CT. Median emergency department length of stay was 8.2 hours (IQR 5.3, 13.5). Sixty-four (73.6%) patients had a normal neurological examination on arrival; eight of these patients (12.5% (95% confidence interval [CI], 5.6–23.2%) had an abnormal cervical spine MRI. Twenty-three patients (26.4%) had an abnormal neurological examination on arrival; two of these patients (8.7%, 95% CI, 1.1–28%) had an abnormal cervical spine MRI. Overall, 10 patients (11.5%) had an abnormal cervical spine MRI. One patient underwent operative intervention due to an unstable injury. Of the remaining nine patients with acute findings on cervical spine MRI, there were no other unstable injuries; two patients were managed with cervical orthosis, and seven patients had no additional management.

**Conclusion:**

In this study of older patients with ground-level falls and normal, atraumatic, cervical spine CT, a small portion had traumatic abnormalities on MRI, with few requiring further intervention. Further study is required to identify criteria to determine when MRI should be performed in older patients after a ground-level fall.

## INTRODUCTION

As the population ages, ground-level falls in older adults are an increasing presentation to emergency departments (ED).^1^,^2^ These visits are costly and often involve extensive diagnostic evaluations.^3^ Evaluating older patients following a ground-level fall with a suspected acute cervical spine injury can be challenging due to pre-existing neurologic deficits, frailty, and cognitive impairment. Furthermore, degenerative and osteoporotic changes frequently occurring in the elderly make cervical spine radiographic interpretations difficult. These factors along with limited research contribute to uncertainty in the appropriate radiologic evaluation of the cervical spine in this population.

Previous studies have evaluated the incidence of positive cervical spine magnetic resonance imaging (MRI) findings after negative cervical spine computed tomography (CT) in the general trauma population with mixed results.^4^ The incidence of clinically significant injuries identified on a cervical spine MRI after a negative cervical spine CT is very low in both alert and obtunded patients.^5^,^6^ Several studies have concluded that the routine use of cervical spine MRI after a negative cervical spine CT is not cost effective and not recommended.^4^,^7^,^8^ Recent studies evaluating the utility of cervical spine MRI after a negative cervical spine CT have focused on the general trauma population with substantially younger patients and all trauma evaluations, and have variably defined clinically significant cervical spine injuries.^9^–^13^ These studies found little benefit in cervical spine MRI after a negative cervical spine CT in the general trauma population; however, results from these studies may not be generalizable to older patients who have fallen. The appropriate imaging pathway for evaluating the cervical spine of older patients with low velocity, ground-level falls remains unknown.

We sought to determine the rate of acute traumatic abnormalities on cervical spine MRI after a normal cervical spine CT in older patients following a ground-level fall. We hypothesized that a cervical spine MRI is low yield and therefore unnecessary in older patients with a normal physical examination on initial, or repeat, physical examination, following a normal cervical spine CT after a ground-level fall.

## METHODS

### Study Design

This was a retrospective, observational cohort study using data from the site’s electronic health record (EHR). The study was approved by the institutional review board.

### Study Setting and Population

The study site is an urban, academic, Level I trauma center. The annual ED volume is approximately 66,000 adult patients. The trauma service admits approximately 3500 patients annually. Inclusion criteria consisted of patients 65 years of age and older who had a ground-level fall with a cervical spine CT without evidence of an acute injury and then underwent sequential cervical spine MRI. Exclusion criteria included interfacility transfers, prisoners, patients without falls, those being evaluated for advanced malignancy or other established pathology, or whose initial CT showed an acute injury.

### Study Protocol

We identified eligible patients from an EHR search for cervical spine MRI orders placed in the ED from May 23, 2017–May 22, 2019. The following elements were directly extracted from the EHR: gender; age; date and time of presentation; and MRI cervical spine order. The EHR was manually reviewed for inclusion and exclusion criteria; 341 patient charts were reviewed, and 87 patients met the final criteria. Manual abstraction of data from the EHR followed the Gilbert methodologic guidelines.^14^,^15^ The primary abstractor was trained prior to data abstraction, and investigators met after abstracting 10 charts for abstraction review. The following elements were manually abstracted from the EHR using a standardized form designed a priori: ground-level fall; trauma team activation; midline cervical spine tenderness; documentation of focal neurological deficit; history of cognitive impairment; altered mental state; evidence of intoxication; Charlson Comorbidity Index including anticoagulation use; CT and MRI reports; hospital admission; outcomes; and cervical spine interventions.^16^ Clinical findings not explicitly stated as present were considered absent. We calculated ED length of stay, Injury Severity Score (ISS) and revised trauma score from data directly and manually abstracted from the EHR.

**Population Health Research Capsule**

What do we already know about this issue?

*Older patients sustain significant cervical spine injuries after ground level falls. The optimal pathway for evaluating the cervical spine of older patients who have fallen is unknown*.

What was the research question?

*Is a negative computed tomography (CT) sufficient to exclude clinically significant injuries in older patients who have fallen?*

What was the major finding of the study?

*Magnetic resonance imaging (MRI) in older patients who have fallen is generally unnecessary after a normal, atraumatic CT scan*.

How does this improve population health?

*MRI after a normal cervical spine CT scan rarely contributes clinically significant information in older patients after a fall and adds time and expense to the emergency department stay*.

Ground-level falls were defined as falls from standing, falls from less than three feet or fewer than five stairs. Imaging studies were considered to be normal if there was no evidence of any acute traumatic injury on the radiology report. We defined an abnormal MRI as any acute traumatic injury including acute fracture, spinal cord injury, or ligamentous injury on MRI report. Patients with any focal neurological finding on initial examination were considered to have an abnormal neurological examination, and patients with no focal neurological findings were considered to have a normal neurological examination.

One reviewer who was blinded to the study’s hypothesis abstracted patient data for all outcomes. An independent reviewer randomly selected 20 charts to measure abstractor reliability. Study data were collected and managed using Research Electronic Data Capture tools (REDCap, Vanderbilt University, Nashville, TN) hosted at the University of California, Davis.^17^,^18^ The primary outcome was any acute traumatic injury identified on the cervical MRI.

### Data Analysis

We desctibe data with simple descriptive statistics. Continuous data are described with the median and interquartile range (IQR). We calculated 95% confidence intervals (CI) where appropriate. Inter-rater agreement for duplicate data abstraction was measured with the kappa statistic.

## RESULTS

A total of 341 older patients underwent cervical spine MRI imaging ordered in the ED during the 24-month study period. This study included 87 patients who met all the inclusion/exclusion criteria (Figure); there were no duplicate encounters. The median age was 74 (IQR 69, 83) years, and 48 (55%, 95% CI, 44–66%) were female. The median ED length of stay was 8.2 (IQR 5.3, 13.5) hours. Overall, 72 patients (82.75%) received a trauma team activation on ED arrival. Indications for cervical spine MRI were not consistently documented in the EHR.

A total of 64 patients (73.6%) presented with a normal neurological examination, and eight (12.5% [95% CI, 5.6–23.2%]) of these patients had an abnormal cervical spine MRI (Table). There were 23 patients (24.6%) presenting with an abnormal neurological examination; two of these patients had an abnormal cervical spine MRI (8.7%, [1.1, 28.0%]). All injuries identified by MRI were ligamentous injuries of the cervical spine.

One patient (1.1%) was ultimately diagnosed with an unstable cervical spine injury and received the highest level trauma activation on arrival to the ED for mild weakness in the upper extremities and severe bilateral lower extremity weakness. Initial cervical spine CT did not show evidence of an acute injury. Cervical spine MRI revealed radiologic evidence of a central cord syndrome with a large C4 disc protrusion with cord compression and edema. The patient subsequently underwent a C3–C6 laminectomy. In the other 22 patients with an initial abnormal neurological examination, the initial focal deficit either resolved or was found to be non-acute/chronic. Inter-rater agreement for duplicate abstraction ranged from kappa = 0.47 (moderate) to 1.0 (perfect).

## DISCUSSION

Despite the large number of older adults who fall and are evaluated in health systems, the best pathway for evaluating potential injuries of the cervical spine of older patients with low-velocity, ground-level falls remains unknown. Extrapolating results from younger trauma patients suffering from all types of mechanisms to older patients after ground-level falls is inappropriate. In our small retrospective sample we found that traumatic abnormalities on cervical spine MRI were uncommon after a normal cervical spine CT, challenging the utility of performing a cervical spine MRI following a normal cervical spine CT. We evaluated a variety of variables, including ambulation prior to arrival, cognitive impairment, initial focal neurological deficit, intoxication, and midline cervical spine tenderness, but none were associated with an abnormal cervical spine MRI. The trauma team activation pathway prioritizes patients receiving anticoagulant medications, and almost all the patients in this study were initially evaluated after trauma team activation. As expected in this older age group, many patients had degenerative changes identified on cervical spine CT probably contributing to cervical spine MRI requests.

One patient presented with clinical evidence of an unstable cervical injury; the cervical spine CT did not show acute injuries, even on repeat radiological interpretation, and the MRI revealed a large C4 disc protrusion with cord compression and edema. The patient ultimately underwent operative stabilization for this injury.

Patients with acute traumatic injuries on cervical spine CT routinely undergo MRI for further injury delineation and evaluation of the spinal cord. In addition, cervical spine MRI continues to be recommended in obtunded patients after a nondiagnostic cervical spine CT if concerns for a cervical ligamentous injury exist.^19^ The EAST trauma practice guidelines for advanced imaging and cervical spine clearance in obtunded trauma patients, however, were recently revised, recognizing that high-quality CT identifies the majority of clinically significant injuries and noting that injuries found only on MRI are of uncertain clinical significance.^20^ This recommendation questions the utility of MRI after a normal CT. Older patients with dementia are usually not obtunded and can identify and communicate tenderness when carefully examined. The process of obtaining a cervical spine MRI in any trauma patient is complicated by prolonged cervical spine precautions, claustrophobia during the scan and, typically, delays in disposition while the MRI is obtained and resulted. Sedation was provided to one-third of this study’s patients during their ED stay, in many instances to facilitate cervical spine MRI. Sedating elderly patients should be avoided when not necessary as complications may occur.

Clinical decision rules can be used to distinguish between those who require advanced imaging and those who do not, but decision rules often exclude older patients.^21^,^22^ Because older patients may experience significant injuries following ground-level falls, caution is warranted, and decision rules may not perform as well in older patients following a fall as they do in younger patients.^23^,^24^,^25^ This has generated concerns that decision rules should be modified to better recognize injury patterns in older adults.^26^,^27^

In the current study, the reasons for an adjunctive cervical spine MRI being ordered after a normal cervical spine CT were not well documented. Almost all our patients received a trauma team evaluation on arrival, as the activation pathway prioritizes trauma patients receiving anticoagulation. Most of the patients were admitted to the hospital, despite low ISS. In this study population, baseline cognitive impairment was uncommon, few patients had an altered mental state, and very few patients were found to be intoxicated. A large minority of patients were documented to have midline cervical spine tenderness. Nearly a quarter of patients had a neurological deficit on initial examination, which was often the initial trauma examination in the resuscitation room, focused on identifying traumatic injuries. Many of these neurological deficits, however, either resolved or were found to be non-acute and did not contribute further to the admission.

There is no literature to support any specific approach to imaging potential cervical spine injuries in older patients with pre-existing neurological deficits such as prior cerebrovascular accidents, and this remains an area of future research. In older patients with ground-level falls, following a normal cervical spine CT, the patient should be carefully re-examined focusing on midline cervical spine tenderness and focal neurological deficits. If these signs and symptoms have resolved, or found to be non-acute, then cervical spine MRI is unlikely to benefit the patient and is not recommended.

## LIMITATIONS

This was a retrospective medical record review and subject to the limitations of this methodology. We followed the Gilbert methodologic guidelines for retrospective medical record review to limit the introduction of bias.^14^,^15^ In addition, this was a single-site study with a small sample size, limiting the generalizability of the findings. Furthermore, indications for cervical spine MRI were not defined and not consistently recorded in the EHR. Patients with similar mechanisms and ages did not all proceed with advanced imaging of the cervical spine, and some of these patients may have had MRI abnormalities if imaged. Although distracting injuries were not specifically identified in this review, all patients were ultimately discharged, and low ISS suggest the absence of other substantial injuries.

## CONCLUSION

In this study of older patients with ground-level falls and normal cervical spine CT, a small portion had traumatic abnormalities on MRI, with very few patients requiring further intervention. Further study is required to identify criteria to determine when cervical spine MRI should be performed in older patients after a ground-level fall.

## Figures and Tables

**Figure 1:**
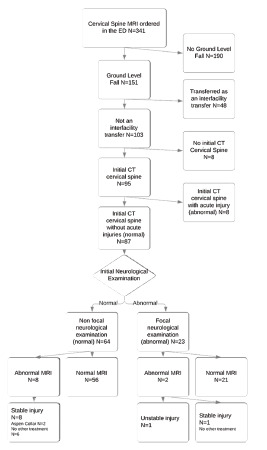
Flow diagram for chart review.

**Table 1:** Patient characteristics of cervical spine magnetic resonance imaging ordered from the emergency department following a ground-level fall.

	MRI without acute injury (n = 77)	MRI with acute injury (n = 10)	Difference in rates/means (95% CI)
Patient characteristics			
Age (years)	76.3	78.2	1.9 (−4.0, 7.7)
Female gender	42 (55%)	6 (60%)	5% (−27, 38%)
Charlson Comorbidity Index	8.2	9.6	1.4 (−0.7, 3.5)
Injury severity score	8.1	10	1.8 (−1.2, 4.9)
Revised trauma score	7.9	8	0.1 (0.1, −0.4)
History			
Cognitive impairment	17 (22%)	2 (20%)	−2% (−29, 24%)
Anticoagulant medications	30 (39%)	6 (60%)	21% (−11, 54%)
Do not resuscitate	11 (14%)	1 (10%)	−4% (−24, 16%)
Ambulatory after fall	25 (32%)	2 (20%)	−12 (−39, 14%)
Physical Examination			
Intoxicated	5 (6%)	1 (10%)	4% (−16, 23%)
Midline C-spine tenderness	29 (38%)	5 (50%)	12% (−20, 45%)
Altered mental status	15 (19%)	0 (0%)	−19% (−28, −10%)
Focal neurological deficit	21 (27%)	2 (20%)	7% (−34, 19%)
ED Evaluation or treatment			
Trauma team activation	62 (81%)	10 (100%)	19% (10, 28%)
Sedatives administered	28 (36%)	1 (10%)	−26% (−47, −5%)
Head CT	70 (91%)	9 (90%)	−1% (−21, 19%)
Interventions			
Operative stabilization	0 (0%)	1 (10%)	10% (−9, 29%)
Cervical orthosis	0 (0%)	2 (20%)	20% (−5, 45%)
Additional C-spine intervention	0 (0%)	0 (0%)	0
Outcomes			
Under-triage	0 (0%)	0 (0%)	0
ED length of stay (hours)	9.7	12.7	3.0 (−1.4, 7.5)
Admission	68 (88%)	9 (90%)	2% (−18, 22%)
Discharged alive	77 (100%)	10 (100%)	0

*MRI*, magnetic resonance imaging; *C-spine*, cervical spine; *ED*, emergency department; *CT*, computed tomography.

Continuous data reported as a mean.

Under triage = Injury Severity Score >16 and no trauma team activation.
